# Association between non-high-density lipoprotein cholesterol-to-high-density lipoprotein cholesterol ratio and macroalbuminuria: evidence from NHANES 1999-2018

**DOI:** 10.3389/fendo.2025.1503780

**Published:** 2025-02-11

**Authors:** Dongli Huang, Yuan He

**Affiliations:** ^1^ Department of Nephrology, Bishan Hospital of Chongqing Medical University (Bishan Hospital of Chongqing), Chongqing, China; ^2^ Department of Infectious Diseases, Bishan Hospital of Chongqing Medical University (Bishan Hospital of Chongqing), Chongqing, China

**Keywords:** the non-high-density lipoprotein cholesterol to high-density lipoprotein cholesterol ratio, macroalbuminuria, NHANES, cross-sectional study, ACR - albumin to creatinine ratio

## Abstract

**Purpose:**

The non-high-density lipoprotein cholesterol to high-density lipoprotein cholesterol ratio (NHHR) is a crucial lipid marker associated with various cardiovascular diseases. However, its relationship with kidney injury, particularly albuminuria, remains poorly understood. This study aims to investigate the association between NHHR and macroalbuminuria in U.S. adults

**Patients and methods:**

This cross-sectional study utilized data from the 1999–2018 National Health and Nutrition Examination Survey (NHANES). NHHR was calculated as (Total cholesterol - HDL cholesterol)/HDL cholesterol. Macroalbuminuria was defined by an albumin-creatinine ratio (ACR) >300 mg/g. Logistic regression, smoothed curve fitting, subgroup analyses, and sensitivity analysis were employed to assess the relationship between NHHR and macroalbuminuria.

**Results:**

A total of 41,225 participants were included in the analysis. Higher NHHR was significantly associated with an increased likelihood of macroalbuminuria (OR = 1.34, 95% CI: 1.13–1.59, p=0.0007). Subgroup analysis revealed a stronger association in participants with BMI ≥30 kg/m^2^(OR = 1.89, 95% CI: 1.44–2.47, p<0.01). Sensitivity analysis revealed that the association remained robust even after excluding participants taking medications that affect lipid metabolism.

**Conclusion:**

In U.S. adults, an increased likelihood of incident NHHR levels of macroalbuminuria is positively associated and is more pronounced in those with a BMI ≥30kg/m^2^.

## Introduction

Increased urinary albumin excretion is not only a marker of early kidney disease. Still, it has also been shown to be an independent predictor of chronic kidney disease (CKD) progression and cardiovascular risk (1). The random urine albumin-to-creatinine ratio (ACR) is widely used to assess and define proteinuria. Macroalbuminuria refers to the presence of elevated levels of albumin in the urine, typically defined as an albumin-to-creatinine ratio (ACR) greater than 300 mg/g. It is considered a key indicator of kidney damage and is commonly associated with chronic kidney disease (CKD), diabetes, and hypertension. Early detection and management of macroalbuminuria are crucial for preventing further renal impairment and cardiovascular complications (2–4). As an important biomarker, macroalbuminuria reflects endothelial dysfunction, glomerular damage, and an increased risk of progressive kidney disease. Because of its significant negative impact on adverse clinical outcomes, proteinuria has become a major public health problem.

In recent years, the ratio of non-high-density lipoprotein cholesterol (NHDL-C) to high-density lipoprotein cholesterol (HDL-C), known as the NHHR, has gained attention as an emerging biomarker. NHHR has been shown to play a significant role in various diseases, including diabetes, depression, osteoporosis, and gallstones (5–8). There is an association between NHHR and chronic kidney disease, negatively correlated with estimated glomerular filtration rate (9). However, its association with proteinuria has not been further investigated. The main objective of this study was to elucidate the relationship between NHHR levels and macroalbuminuria. The findings of this study could enhance the current understanding of NHHR as a potential prognostic indicator for kidney disease progression. This may result in more personalized treatment strategies, particularly for high-risk populations who could benefit from early intervention. Furthermore, the NHHR’s dual association with both kidney disease and cardiovascular risk underscores the importance of addressing the combined effects on renal and cardiovascular health when managing patients with proteinuria, thus fostering a more integrated approach to clinical management.

## Methods

### Study population

**Figure 1:**
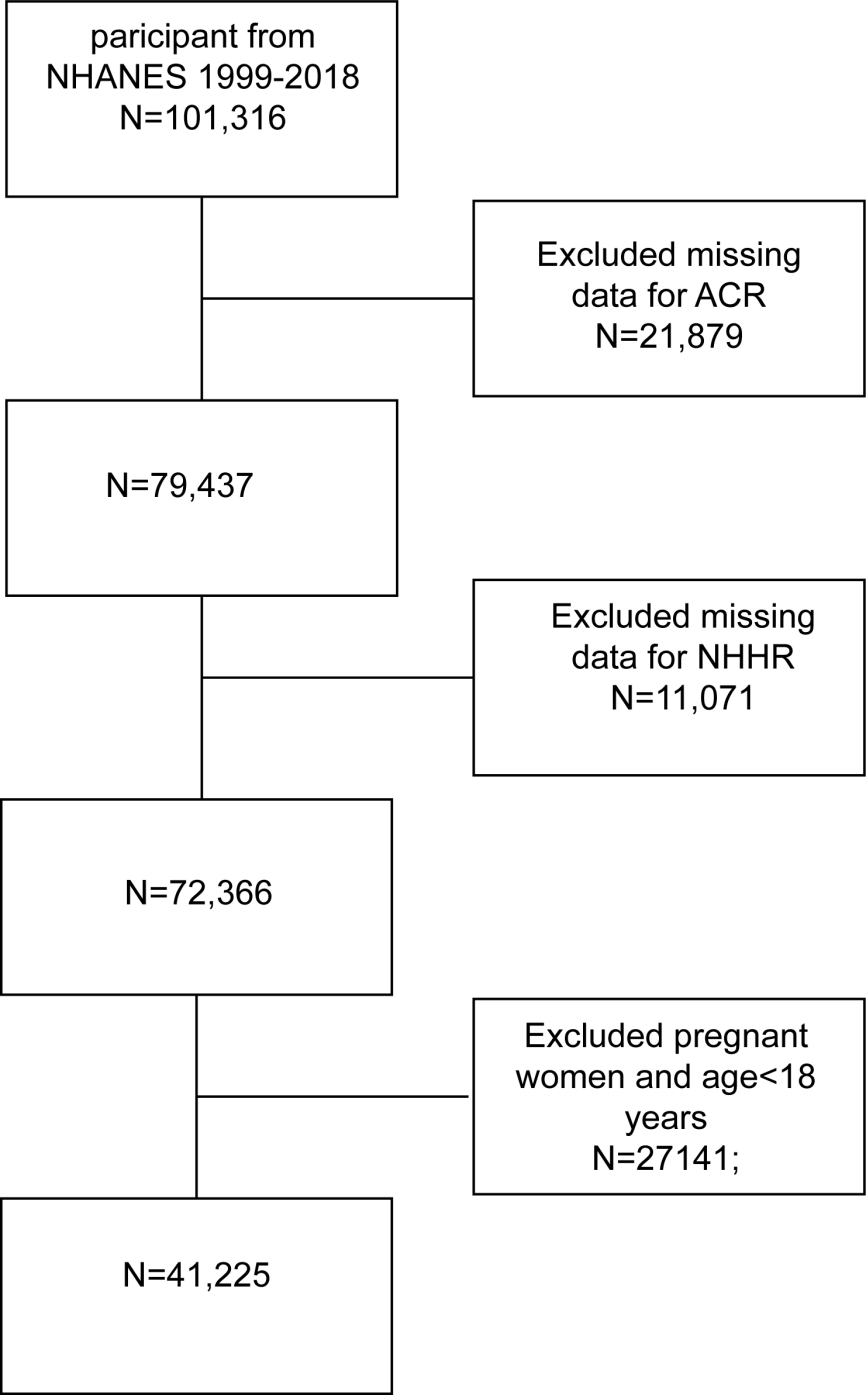
Flowchart of participant selection. NHANES, National Health and Nutrition Examination Survey; NHHR, non-high-density lipoprotein cholesterol to high-density lipoprotein ratio.

The National Health and Nutrition Examination Survey (NHANES), a major initiative by the Centers for Disease Control and Prevention (CDC), aims to evaluate the health and nutritional status of the U.S. population. Our study utilized data from 10 NHANES cycles, spanning 1999 to 2018, including a total of 101,316 participants. After excluding participants with missing data on the albumin creatinine ratio (ACR), total cholesterol, HDL (required for the NHHR formula), pregnant women, and individuals under 18 years of age, the final cohort comprised 41,225 subjects. The inclusion and exclusion process is illustrated in **Figure 1**.

### Study variables

The independent variable in this study was NHHR, calculated as non-HDL-C divided by HDL-C (10). Non-HDL-C is derived by subtracting HDL-C from total cholesterol (TC). Enzyme tests for TC and HDL-C levels were performed using an automated biochemical analyzer. The study used the Roche Cobas 6000 and Roche Modular P chemistry analyzers to measure TC concentrations. These data were obtained directly from the NHANES database.

### Assessment of macroalbuminuria

The dependent variable in this study is the albumin-to-creatinine ratio (ACR). Blood and urine samples from NHANES participants were collected at standardized mobile screening centres. Urinary albumin and creatinine levels were measured using solid-phase fluorescence immunoassay and the modified Jaffe kinetic method with a single-spot urine sample. While modernized for large-scale population studies, this method is rooted in the same scientific principles as the radioimmunoassay approach described by Chavers et al. (11), a historically recognized gold standard for albumin quantification. These measurements were directly retrieved from the NHANES dataset, which utilized automated biochemical analyzers (e.g., Roche Cobas 6000 and Roche Modular P chemistry analyzer) to perform the tests. The ACR was calculated by dividing the urinary albumin concentration (mg) by the urinary creatinine concentration (g). Significant proteinuria was defined as an ACR greater than 300 mg/g (12). In our analysis, macroalbuminuria was treated as an outcome variable.

### Covariates

Covariates potentially influencing the association between NHHR and proteinuria were included in our study (13, 14). These covariates comprised sex (male/female), age (<60 years/≥60 years), ethnicity (Mexican American/Other Hispanic/Non-Hispanic White/Non-Hispanic Black/Other), education level (less than high school/high school or equivalent/college graduate or above), marital status (married/widowed/divorced or separated/never married/living with partner), poverty-to-income ratio (PIR: <1.3, 1.3–3.49, ≥3.5), smoking status (yes/no), alcohol consumption (yes/no), physical activity (yes/no), energy intake(<1500,>=1500 <2500,>=2500kcal), body mass index (BMI: <25, >=25, <30, ≥30 kg/m²), hypertension (yes/no), diabetes mellitus (yes/no), estimated glomerular filtration rate (eGFR: <60/≥60 mL/min/1.73 m²), alanine aminotransferase (ALT, IU/L), aspartate aminotransferase (AST, IU/L), triglycerides (mg/dL), albumin (ALB, g/L), and blood uric acid (UA, mg/dL). Smoking status was defined as having smoked at least 100 cigarettes in a lifetime. Alcohol consumption was defined as having consumed at least 12 alcoholic drinks per year or alcohol ≥3 times in the past 12 months. Diabetes was defined as fasting blood glucose ≥7.0 mmol/L, HbA1c ≥6.5%, having been diagnosed with diabetes by a doctor, currently taking insulin, or using medication to lower blood sugar. Hypertension was defined as systolic blood pressure ≥130 mmHg, diastolic blood pressure ≥80 mmHg, having been diagnosed with high blood pressure by a doctor or currently taking prescription medication for hypertension. Serum creatinine (Scr) was measured using the Jaffe rate method and calibrated by standardized isotope dilution mass spectrometry. eGFR was calculated using the CKD-EPI equation, accounting for participant sex, race, age, and serum creatinine levels (15). Detailed measurement procedures for these variables are publicly available at www.cdc.gov/nchs/nhanes/.

### Statistical analysis

All statistical analyses followed Centers for Disease Control and Prevention (CDC) guidelines, utilizing NHANES sampling weights and accounting for the complex multistage cluster design. Continuous variables were presented as means with standard errors (SE), while categorical variables were expressed as proportions. Weighted Student’s t-tests (for continuous variables) and weighted chi-square tests (for categorical variables) were applied to compare differences across NHHR tertiles. The associations between NHHR and proteinuria were analyzed using three multivariate logistic regression models. Model 1 was unadjusted for covariates. Model 2 adjusted for sex, age, and race. Model 3 adjusted for sex, age, race, education level, marital status, PIR, physical activity, eGFR, ALT, AST, triglycerides, serum uric acid, BMI, hypertension, diabetes, alcohol consumption, and smoking status. Notably, NHHR underwent natural logarithmic (Ln) transformations for regression analysis due to skewed distributions (as shown in **Supplementary Figure 1**). Subgroup analyses of the NHHR-proteinuria association were conducted using stratification factors such as sex (male/female), age (<60/≥60 years), PIR (<1.3/1.3–3.5/>3.5), BMI (<25/>=25, <30/>=30kg/m^2^), hypertension (yes/no), diabetes (yes/no), smoking (yes/no), alcohol consumption (yes/no), eGFR (<60/≥60 mL/min/1.73 m²), energy intake (<1500/>=1500, <2500/>=2500kcal), and physical activity (yes/no). These stratification factors were also considered prespecified potential effect modifiers. Interaction terms were included to assess the heterogeneity of associations across subgroups. Finally, we performed sensitivity analyses; Specifically, we excluded people who used drugs with effects on fat metabolism (statins, steroid hormones, diuretics, beta-blockers) in the last 30 days before performing multivariate logistic regressions. Continuous variables are interpolated using the median, and categorical variables are interpolated using the plurality. All analyses were performed using R version 3.4.3 (http://www.R-project.org, The R Foundation) and Empower software 2.0 version 0 (www.empowerstats.com; X&Y Solutions, Inc., Boston, MA). The significance level was set at p<0.05.

## Results

### Baseline characteristics of participants

41,225 participants met the inclusion and exclusion criteria, with 49.97% being male and 50.03% female. The racial distribution included 45.98% non-Hispanic white, 17.17% Mexican American, 20.08% non-Hispanic black, and 26.39% from other racial/ethnic groups. All participants’ mean ACR and NHHR values were 45.52 mg/g and 3.01, respectively. All clinical characteristics of the participants are presented in **Table 1**, All clinical characteristics of the participants are presented in **Table 1**, categorized by the third quartile of NHHR. Significant differences were observed in age, sex, race, BMI, PIR, smoking status, education, and marital status (*P* < 0.05). Individuals in the highest NHHR tertile were predominantly male and non-Hispanic white. Additionally, those with higher NHHR were more likely to be married and have higher rates of alcohol consumption, elevated triglycerides, and comorbidities such as hypertension, obesity, and elevated ACR.

**Table 1:** Baseline characteristics of NHANES participants, 1999-2018.

Variables	Quartile of Ln-NHHR			*P value*
	Q1	Q2	Q3	
N NHHR	13734 1.67 ± 0.38	13736 2.76 ± 0.32	13755 4.60 ± 1.31	<0.001
ALB, (g/l)	42.46 ± 3.44	42.41 ± 3.38	42.73 ± 3.20	<0.001
UA, (mg/dL)	5.02 ± 1.35	5.46 ± 1.40	5.91 ± 1.43	<0.001
ACR (mg/g)	36.29 ± 250.35	43.02 ± 338.61	57.23 ± 441.14	<0.001
TG, (mg/dL) LDL, (mmol/L)	102.94 ± 38.65 2.37 ± 0.65	126.26 ± 42.23 3.04 ± 0.72	170.84 ± 122.54 3.66 ± 0.92	<0.001 <0.001
HDL, (mmol/L)	1.72 ± 0.41	1.33 ± 0.25	1.04 ± 0.21	<0.001
TC, (mmol/L) RC, (mmol/L)	4.51 ± 0.90 0.42 ± 0.19	4.96 ± 0.91 0.61 ± 0.27	5.68 ± 1.09 0.91 ± 0.38	<0.001 <0.001
ALT, (U/L)	22.03 ± 20.50	24.58 ± 24.72	30.10 ± 29.20	<0.001
AST, (U/L)	25.11 ± 18.80	25.02 ± 20.69	26.69 ± 18.92	<0.001
BMI, n(%)				<0.001
<25	6404 (46.63%)	3727 (27.13%)	2054 (14.93%)	
>=25, <30	4020 (29.27%)	4743 (34.53%)	5186 (37.70%)	
>=30	3310 (24.10%)	5266 (38.34%)	6515 (47.36%)	
Gender, n(%)				<0.001
Male	5114 (37.24%)	6701 (48.78%)	8786 (63.87%)	
Female	8620 (62.76%)	7035 (51.22%)	4969 (36.13%)	
Age, n(%)				<0.001
<60	9001 (65.54%)	8882 (64.66%)	9536 (69.33%)	
>=60	4733 (34.46%)	4854 (35.34%)	4219 (30.67%)	
Race, n(%)				<0.001
Mexican American	1747 (12.72%)	2410 (17.55%)	2922 (21.24%)	
Other Hispanic	943 (6.87%)	1110 (8.08%)	1236 (8.99%)	
Non-Hispanic White	6246 (45.48%)	6254 (45.53%)	6454 (46.92%)	
Non-Hispanic Black	3492 (25.43%)	2776 (20.21%)	2008 (14.60%)	
Other Race	1306 (9.51%)	1186 (8.63%)	1135 (8.25%)	
Education, n(%)				<0.001
Under high school	2985 (21.73%)	3541 (25.78%)	4107 (29.86%)	
Hight school or equivalent	2934 (21.36%)	3220 (23.44%)	3378 (24.56%)	
College graduate or above	7815 (56.90%)	6975 (50.78%)	6270 (45.58%)	
Marital Status, n(%)				<0.001
Married	6537 (47.60%)	7463 (54.33%)	7957 (57.85%)	
Widowed	1286 (9.36%)	1166 (8.49%)	935 (6.80%)	
Divorced	1500 (10.92%)	1452 (10.57%)	1476 (10.73%)	
Separated	465 (3.39%)	458 (3.33%)	439 (3.19%)	
Never married	2934 (21.36%)	2259 (16.45%)	1856 (13.49%)	
Living with partner	1012 (7.37%)	938 (6.83%)	1092 (7.94%)	
PIR, n(%)				<0.001
<1.3	3837 (27.94%)	4162 (30.30%)	4545 (33.04%)	
>=1.3, <3.5	5199 (37.85%)	5248 (38.21%)	5252 (38.18%)	
>=3.5	4698 (34.21%)	4326 (31.49%)	3958 (28.77%)	
eGFR, n(%)				0.322
<60	1206 (8.78%)	1227 (8.93%)	1160 (8.43%)	
>=60	12528 (91.22%)	12509 (91.07%)	12595 (91.57%)	
Diabetes, n(%)				<0.001
Yes	1839 (13.39%)	2216 (16.13%)	2630 (19.12%)	
No	11895 (86.61%)	11520 (83.87%)	11125 (80.88%)	
Drink, n(%)				0.002
Yes	9513 (69.27%)	9309 (67.77%)	9572 (69.59%)	
No	4221 (30.73%)	4427 (32.23%)	4183 (30.41%)	
Hypertension, n(%)				<0.001
Yes	6751 (49.16%)	7676 (55.88%)	8318 (60.47%)	
No	6983 (50.84%)	6060 (44.12%)	5437 (39.53%)	
Physical activity, n(%)				0.787
Yes	5714 (41.60%)	5660 (41.21%)	5707 (41.49%)	
No	8020 (58.40%)	8076 (58.79%)	8048 (58.51%)	
Smoke, n(%)				<0.001
Yes	5853 (42.62%)	6277 (45.70%)	7094 (51.57%)	
No	7881 (57.38%)	6277 (45.70%)	6661 (48.43%)	
Macroalbuminuria,n(%)				<0.001
Yes	239 (1.74%)	283 (2.06%)	358 (2.60%)	
No	13495 (98.26%)	13453 (97.94%)	13397 (97.40%)	
Energy intake, n(%)				<0.001
<1500	4458 (32.46%)	4290 (31.23%)	3824 (27.80%)	
>=1500, <2500	6304 (45.90%)	6231 (45.36%)	6094 (44.30%)	
>=2500	2972 (21.64%)	3215 (23.41%)	3837 (27.90%)	

Categorized according to Ln-NHHR tertiles. NHHR, ratio of non-HDL cholesterol to HDL cholesterol; BMI, body mass index; HDL, high-density lipoprotein cholesterol; TC, total cholesterol; RC, residual cholesterol.

### Association between NHHR and macroalbuminuria

The association between NHHR and macroalbuminuria is presented in **Table 2**. In the unadjusted model, each 1-unit increase in Ln-NHHR was associated with a 51% higher prevalence of macroalbuminuria, and this positive association remained significant after adjusting for all covariates (OR = 1.34; 95% CI, 1.13–1.59, *p*<0.0001). Using tertile 1 as a reference, all three models demonstrated a positive association between NHHR and macroalbuminuria at the T3 level [[Model 1: OR (95% CI) 1.51 (1.28–1.78); Model 2: OR (95% CI) 1.57 (1.33–1.87); Model 3: OR (95% CI) 1.33 (1.10–1.62)], with *p* for trend <0.05 in each model.

**Table 2:** Multiple logistic regression analysis Ln-NHHR vs macroalbuminuria.

	Model 1 OR 95% CI	*P-*value	Model 2 OR 95% CI	*P*-value	Model 3 OR 95% CI	*P*-value
Ln-NHHR	1.51(1.31, 1.75)	<0.0001	1.63(1.40, 1.90)	<0.0001	1.34 (1.13, 1.59)	0.0007
Stratified by Ln-NHHR quartiles						
T1	ref		ref		ref	
T2	1.19 (1.00, 1.41)	0.0523	1.17 (0.98, 1.40)	0.0770	1.11 (0.92, 1.34) 0.2682	0.2796
T3	1.51 (1.28, 1.78)	<0.0001	1.57 (1.33, 1.87)	<0.0001	1.33 (1.10, 1.62)	0.0032
*P* for trend	1.59 (1.32, 1.91)	<0.0001	1.67 (1.38, 2.02)	<0.0001	1.38 (1.12, 1.71)	0.0029

OR: odds ratio.

95% CI: 95% confidence interval.

Model 1: no covariates were adjusted.

Model 2: adjusted for gender, age, and race.

Model 3: gender, age, race, Alb, BMI, education, energy intake, marital status, PIR, UA, eGFR, TG, diabetes, drink, hypertension, physical activity, smoke, ALT, AST.

### A nonlinear correlation between NHHR and macroalbuminuria

**Figure 2:**
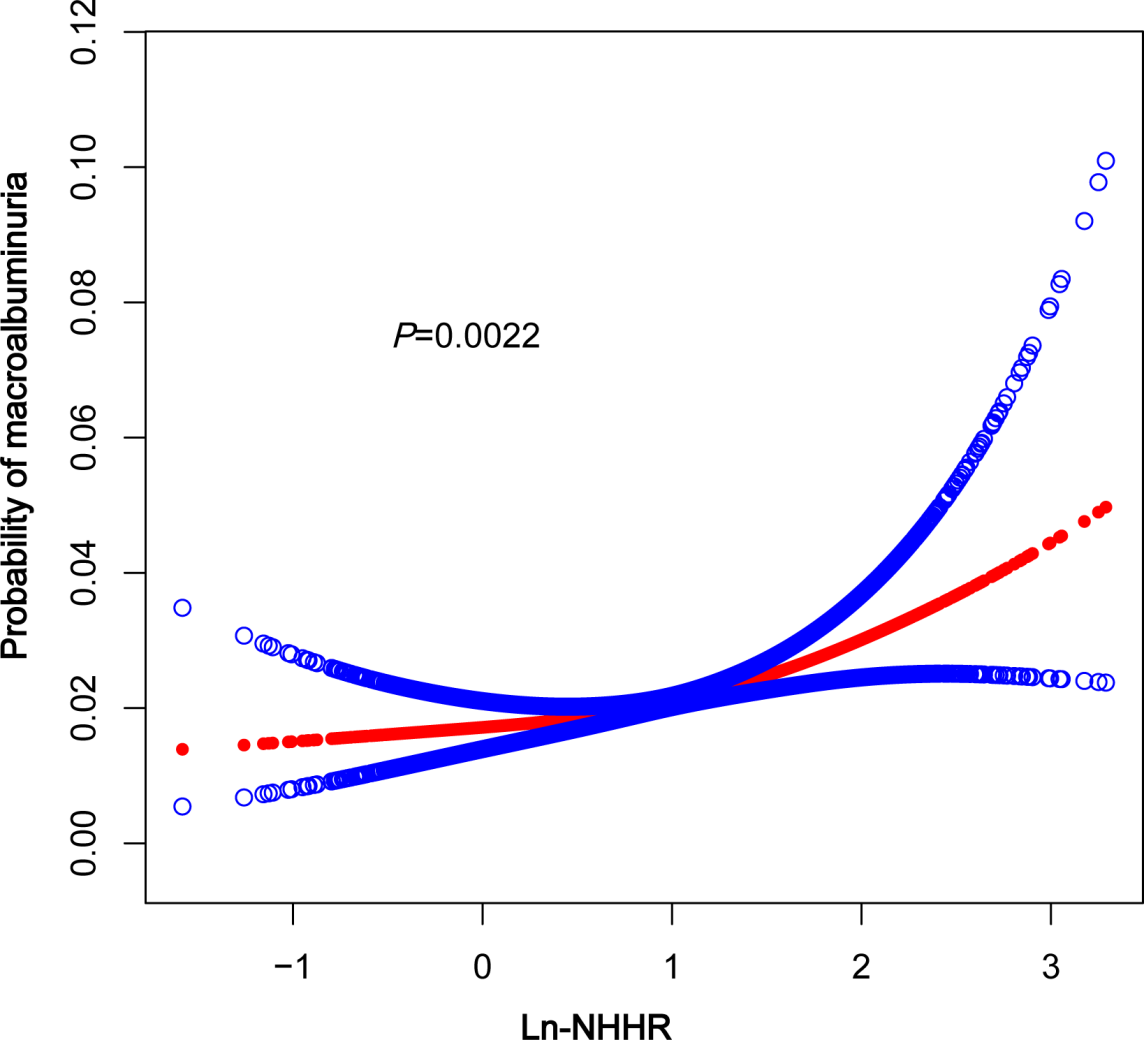
The association between In-NHHR and macroalbuminuria. The solid red line represents thesmooth curve fit between variables, Blue bands represent the 95% confidence interval fromthe fit. NHHR, non-high-density lipoprotein cholesterol to high-density lipoprotein cholesterol.

**Figure 3:**
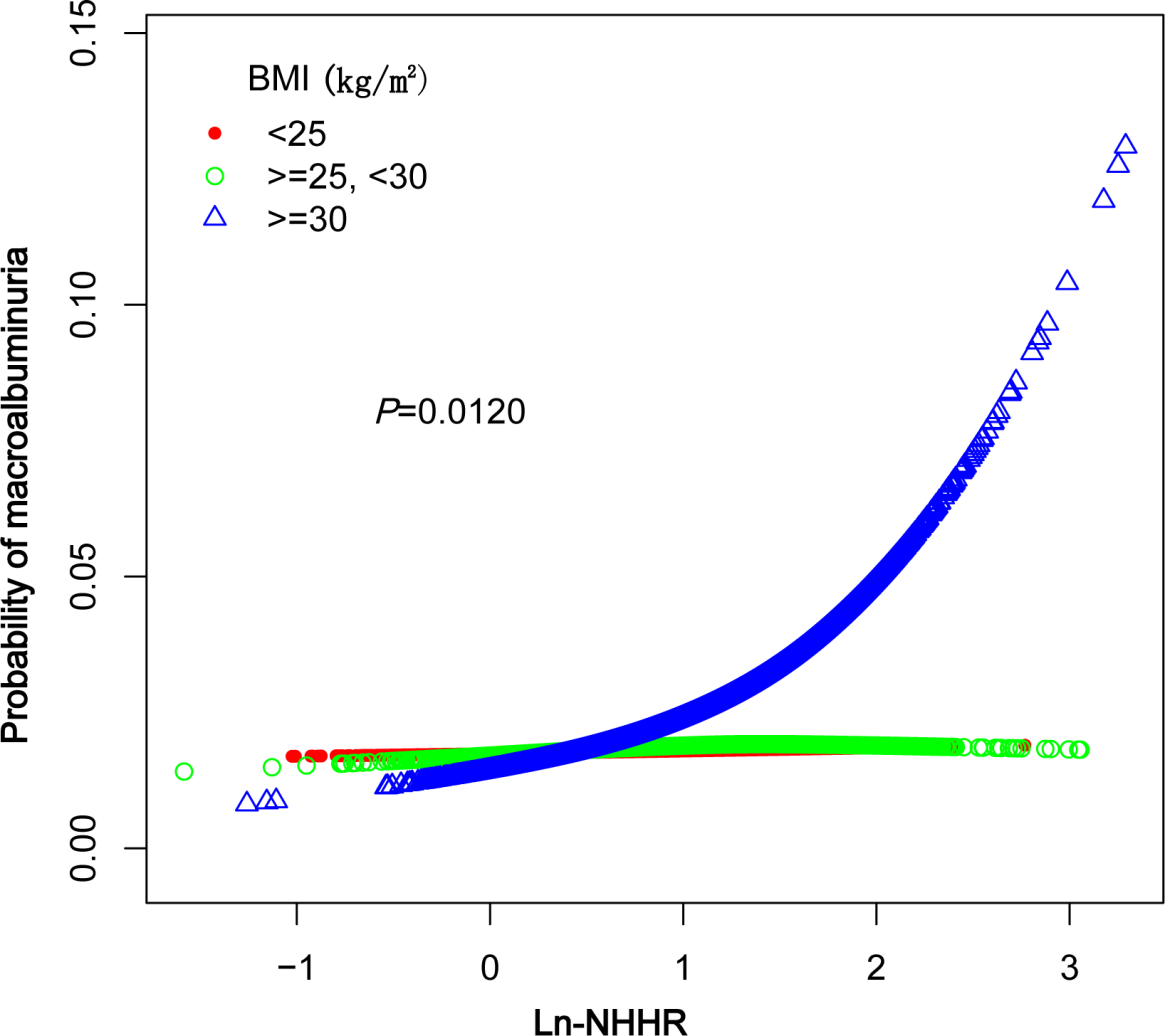
Nonlinear relationship between Ln-NHHR and macroalbuminuria across BMI groups.

A smoothed curve fit was applied to explore the nonlinear relationship between NHHR and macroalbuminuria in greater detail. The results indicated a positive association between NHHR and the prevalence of macroalbuminuria (**Figure 2**). Further subgroup analysis based on BMI (<25, 25–29.9, ≥30kg/m^2^) revealed that this relationship was more pronounced in the BMI ≥30 kg/m^2^ group (**Figure 3**).

### Subgroup analysis

Our subgroup analyses revealed that the association between NHHR and macroalbuminuria was inconsistent. Specifically, the correlation was not statistically significant (p > 0.05) for participants with BMI < 25kg/m^2^, BMI between 25 and 30kg/m^2^, PIR < 1.3, those who did not consume alcohol, and were not diabetic. Additionally, in the BMI subgroups, the odds ratios were: OR = 1.03 (95% CI, 0.74–1.44, p = 0.8593) for BMI < 25kg/m^2^, OR = 1.22 (95% CI, 0.90–1.66, p = 0.1988) for BMI between 25 and 30kg/m^2^, and OR = 1.89 (95% CI, 1.44–2.47, p < 0.0001) for BMI ≥ 30kg/m^2^. A significant difference was observed between groups, with p for interaction = 0.0111. In contrast, sex, age, PIR, eGFR, hypertension, diabetes mellitus, energy intake, smoking, alcohol consumption, and work activity showed no significant effect on this relationship (**Table 3**, p > 0.05 for all interactions).

**Table 3:** Subgroup analysis for the association between NHHR and macroalbuminuria.

Character	OR 95% CI	*P*-value	P for interaction
BMI			0.0120
Blow 25	1.03 (0.74, 1.44)	0.8593	
25-29.99	1.22 (0.90, 1.66)	0.1988	
≥30	1.89 (1.44, 2.47)	<0.0001	
PIR			0.2045
Blow 1.3	1.17 (0.90, 1.53)	0.2442	
1.3-3.5	1.68 (1.24, 2.26)	0.0007	
Over 3.5	1.42 (1.05, 1.92)	0.0232	
Drink			0.1364
Yes	1.54 (1.24, 1.90)	<0.0001	
No	1.19 (0.91, 1.56)	0.2031	
Hypertension			0.5135
Yes	1.36 (1.13, 1.64)	0.0013	
No	1.35 (1.10, 1.65)	0.0035	
Diabetes			0.0911
Yes	1.55 (1.24, 1.94)	0.0001	
No	1.15 (0.89, 1.49)	0.2838	
Work activity			0.5882
Yes	1.51 (1.12, 2.04)	0.0064	
No	1.31 (1.07, 1.60)	0.0095	
Gender			0.9273
Male	1.35 (1.08, 1.69)	0.0083	
Female	1.33 (1.03, 1.72)	0.0287	
Smoke			0.1196
Yes	1.25 (1.01, 1.55)	0.0404	
No	1.59 (1.25, 2.03)	0.0002	
Age			0.8269
<60	1.35 (1.03, 1.77)	0.0307	
>=60	1.40 (1.13, 1.74)	0.0022	
eGFR			0.8229
<60	1.35 (1.03, 1.77)	0.0318	
>=60	1.30(1.04, 1.61)	0.0196	
lipid-lowering treatment			0.9617
Yes	1.33 (1.00, 1.76)	0.0521	
No	1.30 (0.65, 2.59)	0.4518	
energy intake			0.8958
<1500	1.32 (1.01, 1.71)	0.0405	
1500-2500	1.44 (1.11, 1.87)	0.0062	
>2500	1.38 (0.92, 2.08)	0.1196	

### Sensitivity analysis

To address the potential confounding effects of medication use, we conducted a sensitivity analysis by excluding participants who reported using certain prescription drugs that may influence lipid metabolism within one month prior to the NHANES interview. The exclusion criteria were based on the “Prescription Medication” questionnaire, which systematically records medication use. Participants using corticosteroids, diuretics, β-blockers, or statins were excluded. Additionally, information on statin use was further supplemented by the BPQ100D questionnaire. Based on these criteria, we excluded 577 participants using corticosteroids, 5,681 using diuretics, 2,367 using β-blockers, and 3,413 using statins (with some participants using multiple medications). Therefore, the final sample size for the sensitivity analysis was 29,187 participants. To ensure the robustness of our findings, we also performed a multivariate regression analysis. The results from the sensitivity analysis were consistent with the direction of the main analysis (**Supplementary Table 1**).

Furthermore, the original dataset included lipid-lowering treatment records for 8,925 participants (Yes/No), and we conducted an interaction test specifically for these 8,925 participants. The results indicated that lipid-lowering medication use did not affect the association between NHHR and macroalbuminuria, and no significant interaction was observed (**Table 3**).

## Discussion

A cross-sectional analysis of 41,225 U.S. participants indicated that individuals with elevated NHHR were more likely to develop macroalbuminuria. Age, energy intake, and the use of lipid-lowering drugs did not significantly influence this association. However, BMI may significantly influence the strength of the association between NHHR and susceptibility to macroalbuminuria. The prevalence of macroalbuminuria increased more markedly with rising NHHR in individuals with a BMI ≥30kg/m^2^.Furthermore, this relationship was not significant in those without diabetes mellitus.

NHHR is a novel lipid ratio indicator that is relatively low-cost and easy to obtain, with previous studies demonstrating its clinical value across various diseases. A cross-sectional study of the US population found an association between NHHR and diabetic nephropathy, suggesting a potential link to albuminuria (16), which aligns with our findings. However, our study provides a more comprehensive analysis of this association by utilizing macroalbuminuria as a dichotomous outcome variable to elucidate the relationship, focusing on exploring population-specific differences. A cross-sectional study in China revealed that higher NHHR was associated with a higher prevalence of CKD compared to non-HDL-C alone, suggesting that NHHR is a more sensitive predictor of CKD than non-HDL-C (9). Tan found that NHHR is a useful predictor of diabetes risk, with a more pronounced effect in females, laying the foundation for early preventive measures (5). Some studies have also identified a U-shaped association between NHHR and all-cause mortality and an L-shaped association with cardiovascular mortality in U.S. adults with diabetes or prediabetes, suggesting NHHR is a marker of poor prognosis in diabetic patients (10). NHHR has also been linked to prognosis in patients with non-ST-segment elevation myocardial infarction (17). Additionally, studies have revealed associations between NHHR and conditions such as depression, kidney stones, and hyperuricemia (18–20). These studies suggest that NHHR is a significant marker for various diseases, particularly metabolism-related, which aligns with our findings.

The precise mechanism linking NHHR and proteinuria remains unclear. Dyslipidemia, characterized by elevated non-high-density lipoprotein cholesterol (non-HDL-C) and reduced high-density lipoprotein cholesterol (HDL-C), is pivotal in the pathogenesis of proteinuria. Proteinuria patients frequently exhibit lipid metabolism disorders (21). Several hypotheses have been proposed in previous studies, with oxidative stress identified as a critical factor. Non-HDL cholesterol, such as LDL and VLDL, readily oxidizes to form oxidized LDL (ox-LDL) (22, 23). Ox-LDL deposition in the glomerular mesangium and endothelial cell damage may trigger oxidative stress, promoting proteinuria progression (23–25). Additionally, elevated NHHR typically indicates increased LDL levels, while reduced HDL, known for its antioxidant and anti-inflammatory properties (26), may compromise renal protection. This indicates that NHHR might contribute to proteinuria development by amplifying oxidative stress. Additionally, NHHR may exacerbate endothelial dysfunction by promoting the accumulation of atherogenic lipids, significantly influencing the onset and progression of proteinuria (27, 28). Non-HDL lipoprotein accumulation is linked to inflammatory responses, including elevated C-reactive protein and pro-inflammatory cytokines, while reduced HDL exacerbates inflammation by diminishing anti-inflammatory effects (29). Elevated NHHR may serve as an inflammatory marker, contributing to proteinuria via direct glomerular damage or systemic inflammation, including altered renal hemodynamics. Animal studies reveal that mice with non-alcoholic steatohepatitis (NASH) progressively develop podocyte foot process effacement, proteinuria, and renal disease progression, correlating with NASH activity scores (30).

Our subgroup analysis revealed significant between-group differences in BMI (<25, 25–29.9, ≥30kg/m^2^), with the strongest association between NHHR and significant proteinuria observed in the BMI ≥30 kg/m^2^ group (OR: 1.84, 95% CI: 1.40–2.41, P < 0.0001). Obesity, a manifestation of metabolic syndrome, may increase the burden on the kidneys, elevating intraglomerular pressure and accelerating the deterioration of renal function (31). Additionally, obese individuals are more susceptible to lipid metabolism disorders, such as elevated non-HDL cholesterol (non-HDL-C) and decreased HDL cholesterol (HDL-C), which are closely linked to glomerular injury and proteinuria. Obese patients are often characterized by insulin resistance and chronic low-grade inflammation, both of which can exacerbate lipid metabolism disorders and intensify non-HDL-C-induced renal damage in these populations (32, 33). The association between NHHR and significant proteinuria was not significant in individuals with lower BMI (BMI <25kg/m^2^: OR = 1.02, P = 0.9026). This suggests that the association between NHHR and significant proteinuria may be moderated by BMI, with obesity potentially amplifying the effects of NHHR on renal injury. Therefore, BMI may act as an important confounder in the association between NHHR and proteinuria, highlighting the need for stricter lipid metabolism management in obese individuals to minimize renal injury. Additionally, in non-diabetic individuals, the lack of a significant association between NHHR and albuminuria may reflect differences in the pathophysiological drivers of kidney damage. While dyslipidemia plays a central role in diabetic nephropathy, contributing to glomerular injury and oxidative stress (22, 25), non-diabetic albuminuria may arise from other mechanisms, such as inflammatory pathways (32), hemodynamic changes, or genetic factors. These differences may attenuate the impact of NHHR on albuminuria in non-diabetic populations.

This study has several key strengths. Although previous research has explored the relationship between NHHR and albumin-to-creatinine ratio (ACR) (16), it is the first study to use NHHR to examine the relationship between NHHR and macroalbuminuria in the general population using the NHANES database. By employing NHHR as a comprehensive indicator, we offer new insights into the association between abnormal lipid metabolism and kidney damage markers. The data were derived from a nationwide survey in the United States, with a large sample size that provides comprehensive health information across different races, genders, and age groups. Therefore, our findings exhibit strong external validity and can be generalized to diverse populations. Additionally, careful adjustment for confounding variables enhanced the credibility and generalizability of the findings. Finally, the nonlinear relationship between NHHR and clinical proteinuria was explored through smooth curve fitting and subgroup analysis.

However, this study has several limitations. First, as a cross-sectional study, our results demonstrate an association between NHHR and significant proteinuria but do not allow causal inferences. Additionally, the cross-sectional data represent a single time point, limiting our ability to assess the long-term effects of NHHR changes on proteinuria. Future longitudinal studies are necessary to validate our findings. Second, our sample consists solely of US adults, so extrapolating the findings to children or populations in other countries, particularly Asian populations, should be done with caution. Furthermore, although we adjusted for multiple confounders, unmeasured confounders may still exist and could have influenced the relationship between NHHR and proteinuria. Finally, due to the design of the NHANES database, certain exclusion criteria in this study may have introduced selection bias.

## Conclusion

The results of this study indicate a positive correlation between NHHR and the prevalence of macroalbuminuria, particularly in obese patients. Controlling NHHR may have significant clinical implications in preventing the development of proteinuria. However, further prospective clinical trials are necessary to confirm NHHR’s potential role in kidney disease.

## Data Availability

Publicly available datasets were analyzed in this study. This data can be found here: Direct link to the data: The data used in this study were obtained from the National Health and Nutrition Examination Survey (NHANES) and are publicly available from the following link: https://wwwn.cdc.gov/nchs/nhanes/default.aspx Repository name: National Health and Nutrition Examination Survey (NHANES), hosted by the U.S. Centers for Disease Control and Prevention (CDC) National Center for Health Statistics (NCHS). Accession numbers: NHANES datasets are organized by survey years and modules. You can specify the exact survey cycle and dataset names that were used in your study. For example, if you used data from the 2017-2018 cycle, you can mention the specific data files like: NHANES 2017-2018: Demographics Data (DEMO_J), Laboratory Data (e.g., LAB_J). Each dataset in NHANES has its own code (e.g., “DEMO_J” for demographic data), which can be mentioned if applicable.
