# Supplementary figures and images for: Association between non-high-density lipoprotein cholesterol-to-high-density lipoprotein cholesterol ratio and macroalbuminuria: evidence from NHANES 1999-2018
Source: Front Endocrinol (Lausanne). 2025 Feb 11;16:1503780. doi: 10.3389/fendo.2025.1503780 (PMC11851024; doi:10.3389/fendo.2025.1503780)

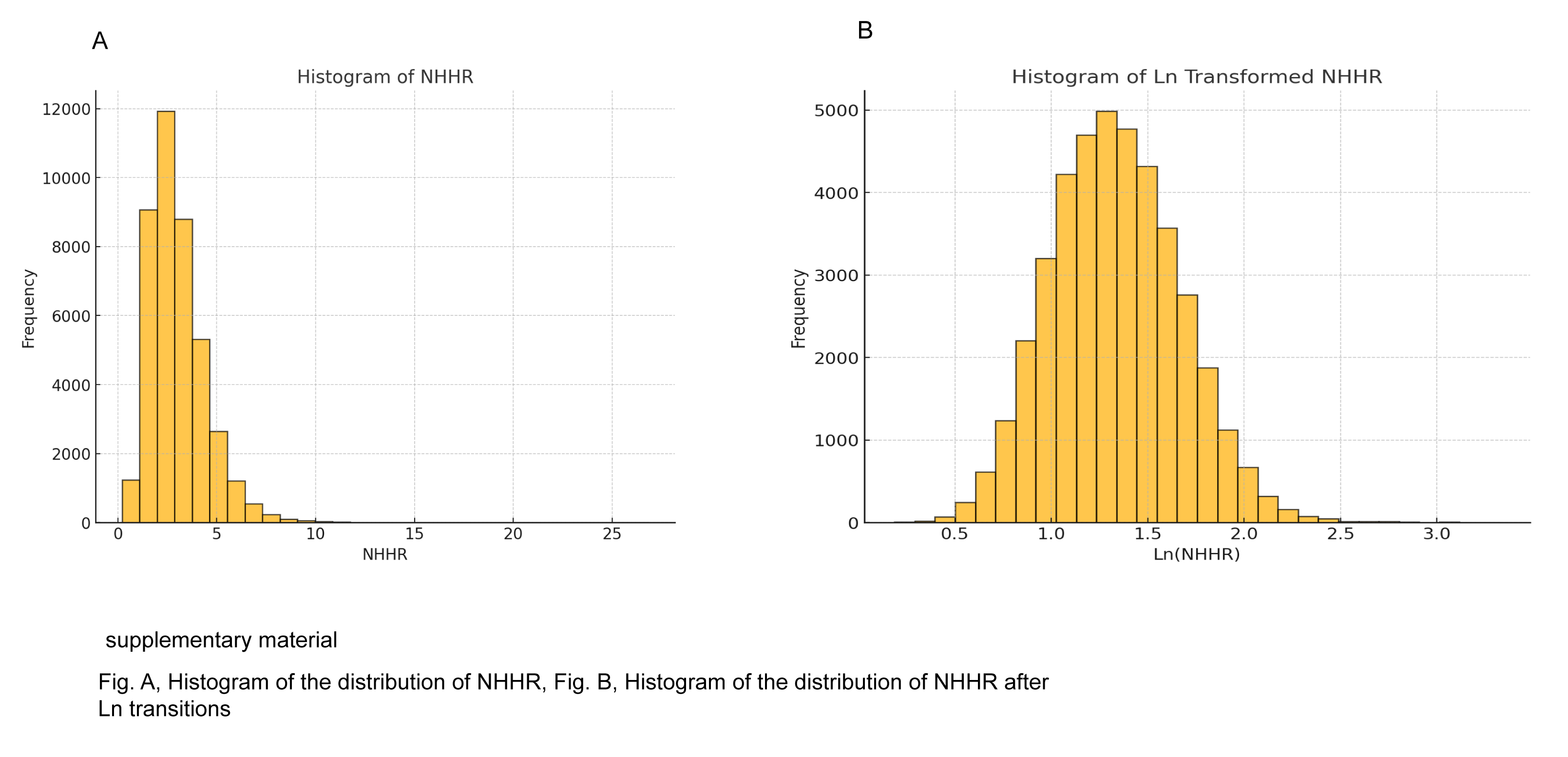

Supplement: Supplementary file 1 [file Image1.tif]

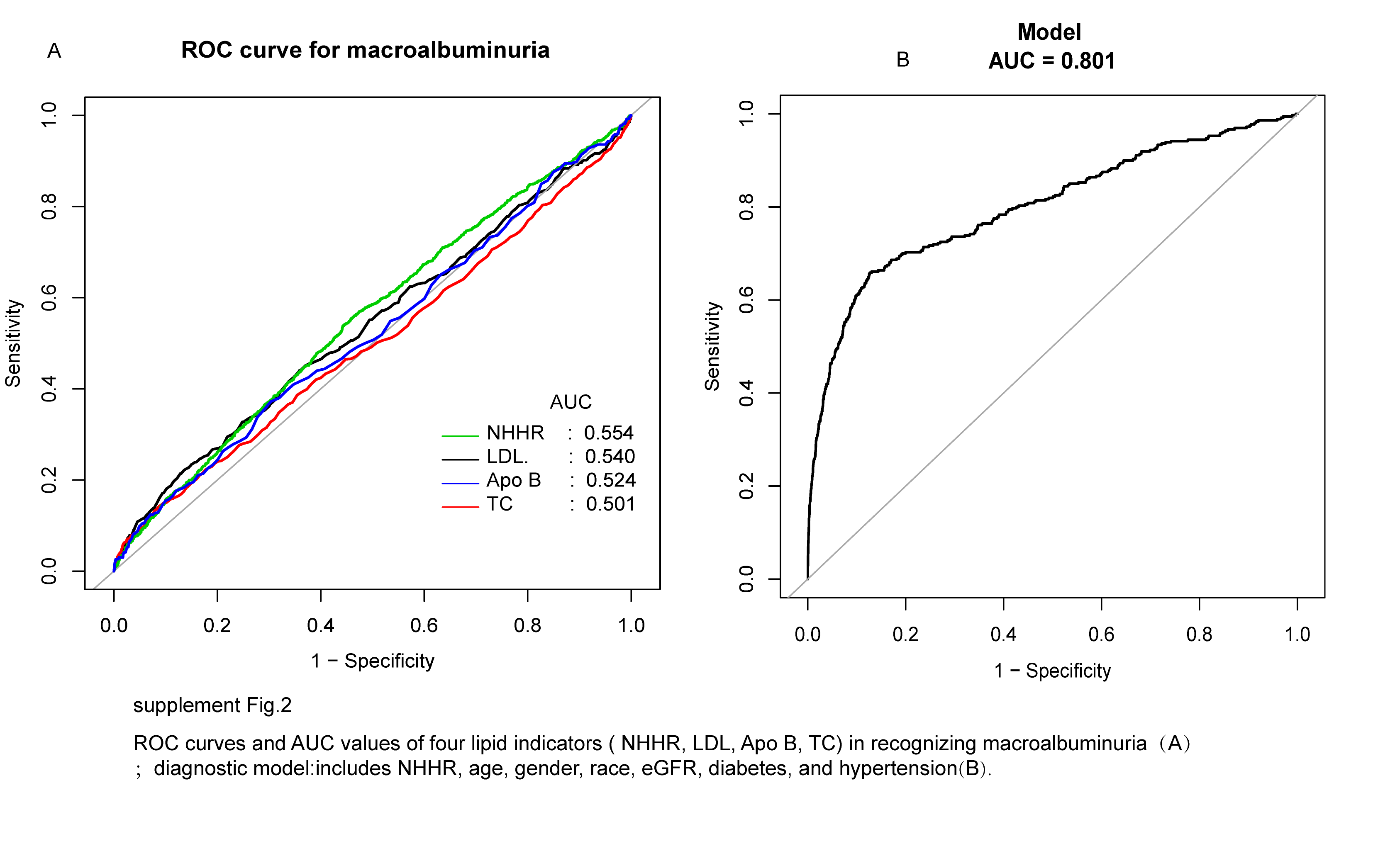

Supplement: Supplementary file 2 [file Image2.tif]
