# Supplementary material for: Association between non-high-density lipoprotein cholesterol-to-high-density lipoprotein cholesterol ratio and macroalbuminuria: evidence from NHANES 1999-2018
Source: Front Endocrinol (Lausanne). 2025 Feb 11;16:1503780. doi: 10.3389/fendo.2025.1503780 (PMC11851024; doi:10.3389/fendo.2025.1503780)
Supplement: Supplementary file 3 [file Table1.docx]

Supplementary Table 1 Multiple logistic regression analysis Ln-NHHR vs macroalbuminuria

|  | **Model 1**  **OR 95% CI** | 1. **value** | **Model 2**  **OR 95% CI** | ***P*-value** | **Model 3**  **OR 95% CI** | ***P*-value** |
| --- | --- | --- | --- | --- | --- | --- |
| Ln-NHHR | 1.66(1.33, 2.08) | <0.0001 | 1.72(1.35, 2.18) | <0.0001 | 1.31(1.01, 1.72) | 0.0048 |
| Stratified by Ln-NHHR quartiles |  |  |  |  |  |  |
| T1 | ref |  | ref |  | ref |  |
| T2 | 1.26(0.96, 1.66) | 0.0969 | 1.21 (0.92, 1.59) | 0.1831 | 1.17 (0.87, 1.57) 0.2682 | 0.2956 |
| T3 | 1.62 (1.25, 2.10) | 0.0003 | 1.60 (1.22, 2.09) | 0.0007 | 1.26 (0.93, 1.70) | 0.1379 |
| *P* for trend | 1.70 (1.28, 2.26) | 0.0002 | 1.68(1.25, 2.26) | 0.0006 | 1.28 (0.92, 1.77) | 0.1423 |

OR: odds ratio

95% CI: 95% confidence interval

Model 1: no covariates were adjusted

Model 2: adjusted for gender, age, and race

Model 3: gender, age, race, Alb, BMI, energy intake, education, marital status, PIR, UA, eGFR, TG, diabetes, drink, hypertension, physical activity, smoke, ALT, AST.

|  |  |  |  |  |  |
| --- | --- | --- | --- | --- | --- |
